# From Incriminating *Stegomyia fasciata* to Releasing *Wolbachia pipientis*: Australian Research on the Dengue Virus Vector, *Aedes aegypti*, and Development of Novel Strategies for Its Surveillance and Control

**DOI:** 10.3390/tropicalmed3030071

**Published:** 2018-06-22

**Authors:** Andrew F. van den Hurk

**Affiliations:** Public Health Virology, Forensic and Scientific Services, Department of Health, Queensland Government, P.O. Box 594, Archerfield, QLD 4108, Australia; andrew.vandenhurk@health.qld.gov.au; Tel.: +61-7-3096-2858

**Keywords:** *Aedes aegypti*, dengue viruses, transmission, Australia, surveillance, control

## Abstract

Globally, the dengue viruses (DENVs) infect approximately 300 million people annually. Australia has a history of epidemic dengue, with outbreaks in the early decades of the twentieth century responsible for tens of thousands of cases. Seminal experiments conducted by Australian scientists during these outbreaks were the first to incriminate *Aedes aegypti* as a major vector of dengue viruses. One hundred years later, Australian scientists are playing a lead role in the development of surveillance and suppression strategies that target this mosquito species. Surveillance of *Ae. aegypti* populations and their associated dengue risk was greatly improved by understanding the contribution of key premises, key containers, and cryptic larval habitats to mosquito productivity, and, more recently, the development of novel adult traps. In terms of mosquito control, targeted indoor residual pyrethroid spraying and community-based biological control utilizing predatory copepods can significantly reduce *Ae. aegypti* populations. The release of *Ae. aegypti* transinfected with the virus-blocking bacterium, *Wolbachia*, provides a promising strategy for limiting DENV transmission. These diverse strategies developed by Australian scientists have the potential to alleviate the burden of dengue in the future, whether it is at the local level or as part of a country-wide program.

## 1. Introduction

Dengue viruses (DENVs) are single-stranded positive-sense RNA viruses globally responsible for an estimated 300 million infections annually [1]. The clinical disease spectrum associated with DENV infection ranges from mild febrile illness through to potentially fatal manifestations, characterized by plasma leakage and shock [2]. There are four DENV serotypes (denoted DENV-1, -2, -3, and -4), which are predominately transmitted between humans via *Aedes aegypti*, although *Aedes albopictus* can be a vector in some instances [3].

Due to the lack of a highly effective vaccine, the primary control strategy for dengue relies on mosquito suppression. This generally involves elimination of water-holding containers that support larval development, and/or application of insecticides targeting larvae and adults. Unfortunately, the effectiveness of control strategies targeting *Ae. aegypti* and *Ae. albopictus* is compromised by the close association between these species and human habitation, which provides numerous containers for larval development, preferred human blood meals, and intradomiciliary feeding and resting sites. Furthermore, incidence of insecticide resistance in populations of *Ae. aegypti* and *Ae. albopictus* is increasing, further impacting the efficacy of mosquito control programs [4].

Whilst dengue imposes its greatest burden on developing countries, outbreaks in Key West (Florida) and northern Australia illustrate the vulnerability of any *Ae. aegypti*-infested region to local DENV transmission [5,6]. Australia has a history of dengue epidemics that stretches back to the late 1800s, although there was a significant increase in the frequency of outbreaks in the last 25 years. Due to the constant threat of DENVs, Australian scientists greatly contributed to the understanding of virus transmission cycles, and developed or refined strategies for *Ae. aegypti* surveillance and control that show considerable promise for DENV control in nations where these viruses exact their greatest toll. This paper revisits early Australian experiments conducted to incriminate *Ae. aegypti* as a DENV vector, then fast forwards approximately 100 years to examine recent advances in our knowledge of *Ae. aegypti* biology and DENV control that Australian scientists performed a lead role in developing.

## 2. Dengue in Australia

**Figure 1:**
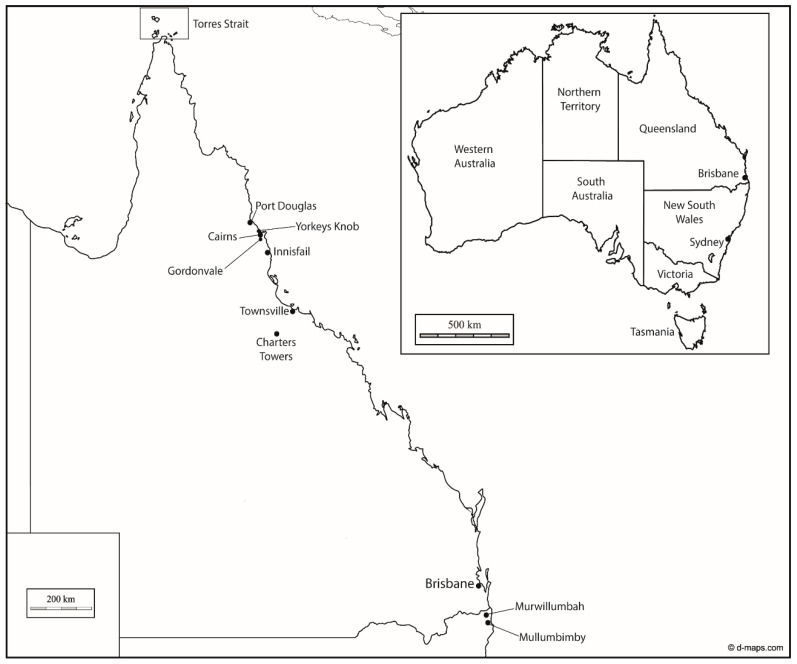
Map of Australia (inset) and Queensland, showing locations mentioned in the text. Murwillumbah and Mullumbimby were the two towns in northern New South Wales where Cleland et al. [25,26,27] collected the mosquitoes that were used in their experiments.

Although the exact timing of introduction is unknown, it is estimated that *Ae. aegypti* was first introduced into Australia in the mid-to-late 19th century [7]. Disease attributed to infection with DENVs was first reported in Australia in the 1870s, and the first widespread outbreak occurred in Queensland in 1897, which included the first formal description of dengue hemorrhagic fever (Figure 1) [8]. This outbreak was followed by another in 1905, which afflicted an estimated 75% of the then population of the state capital, Brisbane, of 125,672, resulting in 94 deaths [9]. Other significant outbreaks occurred in 1916, 1925–1926, and 1942–1943, with each affecting a wide geographical area in northern Australia, and infecting a high proportion of residents in some towns (reviewed in [10]). After a period of little activity, an outbreak of dengue occurred in northern Queensland in 1954–1955, affecting an estimated 15,000 people out of 40,000 in Townsville alone [11].

The latter epidemic was the last to occur in Australia for 26 years. During this time, the geographical distribution of *Ae. aegypti* in Australia contracted considerably. A number of reasons for this range contraction were proposed, including replacement of rainwater tanks by reticulated water, focused public health campaigns to eliminate larval habitats, general improvements in sanitation, and the introduction of household insecticides [12,13]. The current distribution of *Ae. aegypti* in Australia is restricted to Queensland [14], with the closest record to Brisbane being the township of Goomeri, approximately 160 km northwest of the city [15].

Between 1981 and 1983, a widespread outbreak of DENV-1 involving 458 confirmed cases occurred in north Queensland, signaling the return of epidemic dengue to Australia [16]. Local transmission was not recorded for almost a decade, before sporadic locally-acquired cases occurred in 1990–1991 [17,18]. In 1992–1993, outbreaks primarily centered in Townsville and Charters Towers resulted in over 900 reported cases, although retrospective serological studies suggested the number of cases was considerably higher [19,20,21]. A small cluster of DENV-2 cases was reported from a Cairns suburb in 1995 [22]. From that point, a trend to annual outbreaks of dengue occurred in north Queensland, starting with an outbreak of DENV-2 in the Torres Strait in 1996–1997, which was introduced by a resident who had acquired the infection in Papua New Guinea [23,24].

There is no evidence that DENVs are endemic in Australia; rather, outbreaks (defined as one or more locally acquired cases) are initiated by travellers who acquire the infection overseas. The country where the index case acquired their infection is often not conclusively established. However, nucleotide sequencing and phylogenetic analyses of DENVs responsible for 73 outbreaks reported between 1990 and 2017 suggest that the majority originate from the Asia-Pacific region, with viruses from Indonesia accounting for the highest number (23%) of outbreaks, followed by Papua New Guinea (21%) [28]. Outbreaks can involve anywhere from <10 cases to 100s of cases, with intensive vector control activities [29] likely restricting their duration and severity. However, these activities can be overwhelmed by delayed notification of cases (particularly the imported index case [30]), weather conditions that dramatically increase mosquito populations, or the circulation of more pathogenic strains of virus. A perfect storm of these factors contributed to an outbreak of DENV-3 in Cairns in 2008–2009 [6]. Nine hundred and thirty-one cases were reported, making it the largest outbreak recorded in Australia for over 50 years.

## 3. Incrimination of *Ae. aegypti* as a Vector of Dengue Viruses

The seminal experiments conducted by Major Walter Reed and the Yellow Fever Commission in 1900, and work on dengue in Syria by Graham [31], inspired Bancroft [32] to test the hypothesis that the dengue virus was transmitted by mosquitoes to humans. In experiments conducted in Brisbane, locally abundant *Ae. aegypti* (then referred to as *Stegomyia fasciata* [33]) were allowed to feed on patients diagnosed with dengue. After a period of at least 10 days, these mosquitoes were then allowed to bite volunteers, who, in some instances, developed disease typical of dengue. Unfortunately, Brisbane was experiencing a large dengue epidemic at the time, so it was difficult to confirm whether these patients acquired their infection during the experiments or whether infection was naturally contracted. Nonetheless, these experiments were highly suggestive of a role of *Ae. aegypti* in the transmission of DENVs.

To remove any conjecture that the volunteers from Bancroft’s work were not infected experimentally, Cleland et al. [25,26,27] conducted similar experiments in a location free from active DENV transmission. Mosquitoes collected whilst biting infected patients during a DENV outbreak in several towns in northern New South Wales were shipped to Sydney, where there was no evidence of active transmission. These mosquitoes were allowed to feed on volunteers, who were then monitored for signs of dengue disease. The first series of experiments were deemed unsuccessful, reportedly due to the high mortality experienced during transport of the mosquitoes. During the second set of experiments, four of nine subjects developed disease commensurate with DENV infection, and were deemed to be infected by bites from *Ae. aegypti*. On the other hand, there was no evidence of DENV being transmitted by *Culex fatigans* (now known as *Cx. quinquefasciatus*), another common mosquito species that was also present in the mosquito collections.

The authors concluded that, based on their experiments and those of Bancroft [29], *Ae. aegypti* was the most likely vector of DENV in Australia. They also suggested that the geographical distribution of this species coincided with the limits of dengue transmission in northern Australia. In contrast, *Cx. quinquefasciatus* is widely distributed throughout Australia, including regions where local transmission was never reported. If this species was indeed a vector, they hypothesized that local DENV transmission would have occurred where this species was abundant, and where numerous imported cases of dengue were reported. The work of Cleland et al. [25,26,27] incriminating *Ae. aegypti* and discounting *Cx. quinquefasciatus* as DENV vectors was subsequently confirmed by Siler et al. [34] in experiments in Manila, Philippines, in the mid-1920s. Clearly, the pioneering work conducted in Australia in the first decades of the 20th century paved the way for over 100 years of research on *Ae. aegypti*, and formulation of strategies for its control.

## 4. Development of Novel Surveillance and Control Strategies Targeting *Ae. aegypti*

### 4.1. Surveillance of Ae. aegypti Populations

A number of indices are traditionally used to define the level of *Ae. aegypti* infestation and its associated risk for DENV transmission [35,36]. These indices provide a relative estimate of the number of containers positive for *Ae. aegypti* larvae or pupae, with the house, container, and Breteau indices most commonly used. However, the accuracy of these indices is compromised by several factors, most notably the heterogeneous distribution of *Ae. aegypti* in the urban environment, varying container size, utilization of cryptic larval habitats, and inspectors being unable to access all premises in a survey area. Critically, there is little correlation between larval indices and adult densities, and the associated dengue incidence [35].

A comprehensive examination of these larval indices, and the relative contribution of cryptic larval habitats, was conducted in north Queensland by researchers from the Queensland Institute of Medical Research (QIMR) in the 1980s and 1990s. Importantly, they demonstrated that a relatively small number (<10%) of houses accounted for a high proportion (≈50%) of positive containers [37], and designated these highly productive houses as key premises. Similarly, key containers, such as rainwater tanks, wells, and tires, were shown to contribute greatly to *Ae. aegypti* productivity. Other studies revealed that not only were subterranean sites, such as wells, service pits, sump pits, abandoned mine shafts, and drains, highly productive larval habitats in some locations, but they were also linked to DENV exposure, and provided a refuge for *Ae. aegypti* during dry weather conditions [38,39]. Conversely, Montgomery and Ritchie [40] demonstrated that roof gutters could also be highly productive larval habitats. Overall, these studies suggest that during DENV outbreaks, limited resources could be utilized best by targeting key containers and key premises, whilst the contribution of subterranean or elevated sites to productivity should not be underestimated.

Given the issues with immature indices, adult sampling became the recommended paradigm for assessing *Ae. aegypti* populations and subsequent DENV transmission risk [35,36]. Sticky ovitraps were developed and deployed in the 2000s for surveillance of adult *Ae. aegypti* in the Cairns region of north Queensland, providing a tool to assess the relative abundance of gravid *Ae. aegypti* during routine monitoring, as well as complementing lethal ovitrap deployments used for dengue control, and for providing mosquitoes for virus detection [41,42]. However, operational issues with adhesives compromised the deployment of sticky ovitraps in some locations [6]. Whilst it was developed in Germany, some of the early assessment of the efficacy of the Biogents Sentinel (BGS) trap was conducted in Cairns [43,44], and these traps are now used routinely for surveillance of *Ae. aegypti* populations both in Australia and globally [6,45,46]. Although BGS traps are effective for collecting all physiological stages of both sexes of *Ae. aegypti* [43], they are relatively expensive and require a source of electricity. The Gravid *Aedes* Trap (GAT) was developed as an alternative to sticky ovitraps and BGS traps, and represents a relatively inexpensive trap for monitoring adult *Ae. aegypti* [47,48], including males that can be attracted by sound lures broadcasting female flight tones [49].

Given the former presence of *Ae. aegypti* in Brisbane and the constant threat of *Ae. albopictus* being introduced, considerable resources are required to confirm the continued absence of these species in southeast Queensland. Deployment of adult traps in extensive arrays over a large spatial scale during presence–absence surveys can be expensive and logistically challenging. Ovitraps provide an inexpensive and sensitive trap alternative. However, the time and laboratory resources required to rear and identify larvae compromises their efficacy. To expedite presence–absence surveys over a large geographical area, Montgomery et al. [15], developed the Rapid Surveillance for Vector Presence (RSVP), which integrates ovitraps, egg quantification, and molecular identification of newly-hatched first instar larvae. The molecular assays developed for RSVP can detect a single first instar *Ae. aegypti* larva in a pool of 5000 non-target first instar larvae, so eggs from multiple ovitraps can be pooled for each diagnostic test, thus reducing processing time.The RSVP is now implemented operationally by local governments in southeast Queensland, and the utility of citizen science for increasing the number of ovitrap collections is currently being evaluated (https://metrosouth.health.qld.gov.au/zika-mozzie-seeker; accessed on 30 May 2018). For this pilot project, residents deploy ovitraps for a two-week period before posting ovistrips to a central diagnostic facility for analysis.

### 4.2. Evolution of Insecticide-Based Methodologies

In the early 1990s, control of *Ae. aegypti* in north Queensland by health authorities primarily relied on source reduction and treatment of larval habitats with the organophosphate insecticide, temephos, as well as occasional supplementation with outdoor ultra-low volume (ULV) application of pyrethroid and organophosphate insecticides [50]. Indeed, these strategies still form the mainstay of DENV control in many parts of the world [51]. In 1995, the insect growth regulator *s*-methoprene was first introduced into dengue control operations in north Queensland during a small outbreak of DENV-2 in Cairns ([22], S. Ritchie, personal communication). When compared with other insecticides, *s*-methoprene has limited non-target effects, pellet and briquette formulations provide residual control, and there is little evidence of resistance in target mosquito species [52].

During a widespread outbreak of DENV-2 in the Torres Strait in 1997–1998 [24], it soon became evident that source reduction and treatment of larval containers alone was not sufficient to stop transmission. It was decided that adult female *Ae. aegypti* needed to be targeted indoors, where it was biting and resting. Thus, a team was dispatched to Erub Island to apply the residual pyrethroid insecticide, deltamethrin, within houses using a portable ULV space sprayer. This method was then used on several other islands, and possibly limited transmission on islands where this indoor fogging was applied. Similar application of insecticides, this time with the over-the-counter residual pyrethroid, cypermethrin, was used to supplement larval control strategies when transmission expanded to Cairns [24].

Between 1997 and 1999, an outbreak of DENV-3 centered in Cairns caused almost 500 cases, with 20% of notified cases hospitalized [53]. It was during this outbreak that indoor surface spraying using the residual pyrethroids, deltamethrin and lambda-cyhalothrin, was integrated into emergency control strategies to interrupt transmission. This targeted indoor residual spraying (TIRS; [54]) consisted of spraying indoor resting sites of *Ae. aegypti*, such as under tables, inside cupboards, or any other dark location, with aqueous formulations using pneumatic sprayers; control generally lasted four to eight weeks. Another weapon was added to the dengue control arsenal in north Queensland in 2003, when intensive lethal oviposition trap (ovitrap) arrays, similar in design to those trialed in Brazil and Thailand [55,56], were operationally deployed as part of a response to a DENV-2 outbreak [57].

Recent analyses conducted by Vazquez-Prokopec et al. [54,58] on outbreaks of DENV-2 and DENV-3 in Cairns in 2003 and 2008–2009, respectively, revealed that TIRS integrated with contact tracing of notified dengue cases and larval control can lead to a significant decrease in the probability of future DENV transmission. Whilst this strategy was undoubtedly successful in north Queensland for focal outbreaks, it is very resource- and labor-intensive. Thus, it may be difficult to implement TIRS during intense epidemics in regions with poorly-developed public health systems. Instead, Vazquez-Prokopec et al. [54] suggested that prophylactic TIRS, coupled with source reduction, could be implemented prior to the transmission season, and focused on at-risk neighborhoods, such as those with a history of DENV outbreaks.

Of significance, it was suggested that the current pyrethroid susceptibility of Australian *Ae. aegypti* populations is due to the use of strategies that employ different modes of action. Larvae are controlled using the insect growth regulator, *s*-methoprene, while synthetic pyrethroids are used to kill adults using TIRS and lethal ovitraps [59]. This contrasts with *Ae. aegypti* populations from other countries, where widespread indiscriminate application of adulticides (e.g., outdoor ULV space spraying) and larvicides with the same mode of action, led to high levels of pyrethroid resistance [4].

### 4.3. Predatory Copepods as a Key Component of a Community-Based Dengue Control Strategy in Vietnam

The considerable burden of dengue in Vietnam prompted the formulation of a community-based strategy to reduce populations of *Ae. aegypti*. Australian scientists provided expert technical advice to their counterparts in Vietnam, whilst considerable funding was provided by the Australian Government and United Kingdom’s foreign aid programs. The strategy consisted of 4 main elements: (a) community engagement, with support provided by various levels of government; (b) prioritization of control resources based on mosquito productivity from key containers; (c) deployment of predatory copepods of the genus *Mesocyclops* to consume *Ae. aegypti* larvae in water storage vessels; and (d) community participation in various elements of the control program [60].

This strategy was a departure from government-administered programs which historically struggled to provide long-term control of *Ae. aegypti*. Instead, much of the responsibility was transferred to the communities, whilst containers that produced the majority of *Ae. aegypti* were targeted, meaning that resources could be deployed where they would have the greatest impact. Previous field trials demonstrated the utility of *Mesocylops* in reducing larval mosquito populations in large containers, wells, and even abandoned mineshafts, with much of the work conducted by, or in collaboration with, scientists from QIMR [61,62,63]. As part of the Vietnam program, local species of *Mesocyclops* were mass-reared, posted to target communities, and then inoculated into large water storage tanks that were prolific producers of *Ae. aegypti*. *Mesocyclops* and *Ae. aegypti* populations were routinely monitored post-inoculation using a variety of sampling methods [64,65]. During a pilot field trial, *Ae. aegypti* was eliminated from Phanboi village, Haihung Province, northern Vietnam, a village of 400 houses [66]. Elimination only occurred when smaller containers that were not suitable for *Mesocyclops* treatment were removed as part of a community-wide recycling program. The program was expanded into a number of other provinces, leading to the elimination or suppression of *Ae. aegypti*, and a concomitant decrease in DENV infections [60,67,68]. Indications are that the program is sustainable at least seven years after the conclusion of formal control activities, and it appears to be a cost-effective strategy of controlling *Ae. aegypti* in Vietnam [69].

### 4.4. Utilizing Wolbachia for Dengue Control

A number of emerging technologies hold considerable promise for the sustained reduction in DENV transmission or elimination of *Ae. aegypi* populations (reviewed in [70]). In terms of progression to field releases, *Wolbachia*-based strategies are the most advanced of these technologies [71]. *Wolbachia* is an intracellular bacterium that occurs in over 60% of insect species [72]. High rates of maternal transmission, coupled with cytoplasmic incompatibility (CI), allow *Wolbachia* to rapidly drive into target insect populations. The process of CI gives infected insects a reproductive advantage, because crosses between uninfected females and infected males produce no offspring. However, crosses between infected females and uninfected or infected males produce viable offspring, allowing *Wolbachia* to perpetuate in the population.

*Wolbachia* naturally infects some medically important mosquito species, such as *Ae. albopictus* and *Cx. quinquefasciatus*. In contrast, *Ae. aegypti* is not naturally infected with *Wolbachia*; instead, the bacterium is transinfected from a natural host, such as *Drosophila melanogaster* or *Ae. albopictus*. Transinfection involves taking the bacterium from the natural host and microinjecting it into the eggs of *Ae. aegypti*. This was an extremely laborious process, which resulted in an exceedingly high level of mortality in the transinfected line. Using methods of Xi et al. and McMeniman et al. [73,74], McMeniman et al. [75] was able to generate a line of *Ae. aegypti* stably infected with the *w*MelPop strain of *Wolbachia*. The *w*MelPop strain reduces the lifespan of *D. melanogaster*, and it was proposed that if this strain was successfully transinfected into *Ae. aegypti*, then it would reduce the lifespan of this species. A reduction in the lifespan would mean that fewer *Ae. aegypti* would survive for the length of the extrinsic incubation period, which is the time from when a virus is ingested by a mosquito in an infectious blood meal until it can be transmitted. Not only did *w*MelPop reduce the lifespan of transinfected mosquitoes in small cage trials [75], but it also conferred other phenotypes on *Ae. aegypti*, including altered blood-feeding behavior, and decreased fecundity and egg desiccation resistance [76,77].

It was previously shown that *Wolbachia* afforded *D. melanogaster* protection from the infection with the highly pathogenic Drosophila C, cricket paralysis, and Flock House viruses [78]. Consequently, a number of experiments were undertaken at several laboratories in Brisbane, which revealed that DENV-2 and chikungunya virus replication and transmission were significantly inhibited by *Wolbachia* in transinfected *Ae. aegypti* [79]. So, rather than *Wolbachia* being used to change the age structure of *Ae. aegypti* populations, the focus shifted to releasing *Wolbachia*-infected mosquitoes to prevent these mosquitoes from transmitting DENVs [80]. However, the fitness costs associated with *w*MelPop meant that *Ae. aegypti* infected with this strain would struggle to survive post-release, especially during periods of low rainfall, such as the dry season in tropical locations [81]. Consequently, another strain of *Wolbachia* from *D. melanogaster*, *w*Mel, was transinfected into *Ae. aegypti*, where it displayed potent virus-inhibiting properties without the deleterious fitness costs on the host [82]. In purpose-built semi-field cages constructed near Cairns [83], *w*Mel rapidly invaded *Ae. aegypti* cohorts, and reached fixation as early as 30 days after weekly releases commenced [82].

Before proceeding to field deployment, extensive community engagement was undertaken and biosafety approval was obtained from the Australian Pesticides and Veterinary Medicines Authority. In early January 2011, weekly releases of *w*Mel-infected adult *Ae. aegypti* commenced in the north Queensland towns of Gordonvale and Yorkeys Knob [80]. Over 300,000 *w*Mel-infected mosquitoes were released during a 10-week period, resulting in 80%–90% infection frequency. Continual monitoring revealed that high *Wolbachia* frequency was maintained in the populations since this time [84]. Importantly, the virus-blocking phenotype was retained for at least one year post-release [85], and indications from Vietnam suggest longer-term stability [86]. Over the next six years, the World Mosquito Program (formerly Eliminate Dengue) conducted releases across a number of cities and towns in far north Queensland, including Cairns, Townsville, Charters Towers, Innisfail, and Port Douglas (http://www.eliminatedengue.com/australia, accessed on 30 May 2018). Similar releases were attempted with *w*MelPop-infected *Ae. aegypti* in north Queensland and Vietnam, but the associated fitness costs prevented the establishment of this *Wolbachia* strain in these populations [87]. Paradoxically, it was suggested that the lack of desiccation resistance in *w*MelPop-infected mosquito eggs could be exploited to eliminate focal populations of *Ae. aegypti* [88].

The success of releases in north Queensland led to the World Mosquito Program establishing deployment programs in 12 countries, including Brazil, Indonesia, Vietnam, and four countries of the southwestern Pacific region (http://www.eliminatedengue.com/program, accessed on 30 May 2018). Whilst it undoubtedly drives to near fixation in local *Ae. aegypti* populations, the success of *Wolbachia*-based interventions will be measured by a reduction in disease incidence attributed to DENV infection. Several different approaches are being used to evaluate the impact of *Wolbachia*, and include cluster randomized trials and observational studies, whereby the impact on dengue incidence is tracked through time (summarized in [89,90]). The results of these epidemiological assessments will start becoming available over the next several years [91].

Like any biocontrol strategy, there are potential vulnerabilities in *Wolbachia*-based interventions [71]. Vulnerabilities that could impact effectiveness include the loss of or attenuation of *Wolbachia* infection in the mosquito, and the emergence of virus strains that are not only resistant to *Wolbachia*-mediated blocking, but which also increase virulence and disease pathogenesis in humans. Another issue that has been raised is the possible enhancement of the arbovirus infection in transinfected mosquitoes. This was based on a study that demonstrated that transient infection of *Cx. pipiens* with the *w*AlbB strain of *Wolbachia* resulted in an enhancement of a subsequent West Nile virus infection [92]. However, the authors acknowledged that their results may have differed if a stably-transinfected line of *Cx. pipiens* was used, instead of a transiently-infected line. Nonetheless, these potential vulnerabilities emphasize the complexity of *Wolbachia*–virus–mosquito interactions and the need for a thorough assessment of candidate strains to identify any risks before being considered for release. Importantly, ongoing monitoring of transinfected populations will be critical to ascertain any changes in *Wolbachia* prevalence and tissue density, whilst vector competence experiments will ensure the virus-blocking phenotype is being maintained [71]. To limit the impact of these confounding factors, *Ae. aegypti* infected with a different *Wolbachia* strain or superinfected with multiple strains could be deployed [93,94].

## 5. Conclusions and Future Perspectives

Although there was some spectacular success in eliminating *Ae. aegypti*, most notably in South and Central America during the middle of the 20th century [95], the ever-increasing global burden of DENVs indicates that elimination or larvicide treatment of containers, and indiscriminate application of adulticides are largely ineffective for sustained control of *Ae. aegypti*. Anthropogenic factors, including unchecked urbanization and lack of associated infrastructure, proliferation of containers that support larval development, and poorly-resourced public health systems, further compromised the efficacy of these control strategies. Indeed, the increase in frequency of dengue outbreaks in north Queensland since the late 1990s necessitated that control strategies moved to a model that integrated patient contact tracing, TIRS, and larval control. Whilst these strategies undoubtedly limited the scale of DENV outbreaks in Australia [54,58], they are resource-intensive, expensive, and can occasionally be overwhelmed, such as during the 2008–2009 Cairns DENV-3 outbreak.

The widespread deployment of *Wolbachia* since 2011 altered the dynamics of the relationship between *Ae. aegypti* and DENVs in north Queensland. Should the *Wolbachia* approach be as effective as modeling predicts in reducing transmission [89], then limitations of current control practices may not be as pronounced. Although there were fewer notified locally-acquired cases of dengue in the most recent years when compared with preceding years [96], the historical number of locally acquired cases in north Queensland is insufficient to statistically demonstrate a causative effect of *Wolbachia* on reduced local DENV transmission. Instead, the epidemiological studies currently underway in hyperendemic locations should provide the crucial data on the impact of *Wolbachia* on dengue incidence in a field setting. In response to the widespread deployment of *Wolbachia* in north Queensland, health authorities are integrating *Wolbachia* monitoring in *Ae. aegypti* populations into routine surveillance and control operations. For the foreseeable future, all suspected dengue cases will continue to be investigated, and the capacity to conduct intensive vector control activities based on TIRS and larval control will be maintained.

Unfortunately, *Ae. aegypti* is an adversary that continues to defy all efforts of controlling it. In the future, an integrated approach to surveillance and control, including source reduction, targeted insecticide application, and continued judicious deployment and monitoring of *Wolbachia* will have the greatest impact on mosquito populations and the associated the burden of DENVs. However, there are numerous issues that will need to be addressed as novel surveillance and control strategies are rolled out across highly urbanized regions where DENVs exact their greatest toll. The most important of these are their scalability and sustainability, as well as their impact on dengue incidence. Regardless, the considerable contribution that they made since Bancroft first incriminated *Ae. aegypti* as a vector of DENVs suggest that Australian scientists will be at the forefront of the development and adoption of these strategies.

## References

[1] Bhatt S., Gething P.W., Brady O.J., Messina J.P., Farlow A.W., Moyes C.L., Drake J.M., Brownstein J.S., Hoen A.G., Sankoh O. (2013). The global distribution and burden of dengue. Nature.

[2] World Health Organization (2009). Dengue: Guidelines for Diagnosis, Treatment, Prevention and Control—New Edition.

[3] Carrington L.B., Simmons C.P. (2014). Human to mosquito transmission of dengue viruses. Front. Immunol..

[4] Moyes C.L., Vontas J., Martins A.J., Ng L.C., Koou S.Y., Dusfour I., Raghavendra K., Pinto J., Corbel V., David J.P. (2017). Contemporary status of insecticide resistance in the major *Aedes* vectors of arboviruses infecting humans. PLoS Negl. Trop. Dis..

[5] Radke E.G., Gregory C.J., Kintziger K.W., Sauber-Schatz E.K., Hunsperger E.A., Gallagher G.R., Barber J.M., Biggerstaff B.J., Stanek D.R., Tomashek K.M. (2012). Dengue outbreak in Key West, Florida, USA, 2009. Emerg. Infect. Dis..

[6] Ritchie S.A., Pyke A.T., Hall-Mendelin S., Day A., Mores C.N., Christofferson R.C., Gubler D.J., Bennett S.N., van den Hurk A.F. (2013). An explosive epidemic of DENV-3 in Cairns, Australia. PLoS ONE.

[7] Lee D.J., Hicks M.M., Griffiths M., Russell R.C., Marks E.N. (1987). The Culicidae of the Australasian Region: Entomology Monograph No. 2. Vol. 4.

[8] Hare F.E. (1898). The 1897 epidemic of dengue in north Queensland. Aust. Med. Gaz..

[9] British Medical Association (Queensland Branch) (1905). Report on the dengue epidemic in Brisbane in 1905. Aust. Med. Gaz..

[10] Lumley G.F., Taylor F.H. (1943). Dengue.

[11] Rowan L.C. (1956). An epidemic of dengue-like fever, Townsville, 1954: Clinical features, with a review of the literature. Med. J. Aust..

[12] Russell R.C., Currie B.J., Lindsay M.D., Mackenzie J.S., Ritchie S.A., Whelan P.I. (2009). Dengue and climate change in Australia: Predictions for the future should incorporate knowledge from the past. Med. J. Aust..

[13] Trewin B.J., Darbro J.M., Jansen C.C., Schellhorn N.A., Zalucki M.P., Hurst T.P., Devine G.J. (2017). The elimination of the dengue vector, *Aedes aegypti*, from Brisbane, Australia: The role of surveillance, larval habitat removal and policy. PLoS Negl. Trop. Dis..

[14] Beebe N.W., Cooper R.D., Mottram P., Sweeney A.W. (2009). Australia’s dengue risk driven by human adaptation to climate change. PLoS Negl. Trop. Dis..

[15] Montgomery B.L., Shivas M.A., Hall-Mendelin S., Edwards J., Hamilton N.A., Jansen C.C., McMahon J.L., Warrilow D., van den Hurk A.F. (2017). Rapid surveillance for vector presence (RSVP): Development of a novel system for detecting *Aedes aegypti* and *Aedes albopictus*. PLoS Negl. Trop. Dis..

[16] Kay B.H., Barker-Hudson P., Stallman N.D., Weimers M.A., Marks E.N., Holt P.J., Muscio M., Gorman B.M. (1984). Dengue fever, reappearance in northern Queensland after 26 years. Med. J. Aust..

[17] Centre for Arbovirus Reference and Research, Queensland Health Department—Queensland University of Technology (1991). Arbovirus activity in Queensland. Commun. Dis. Intell..

[18] Phillips D., Aaskov J. (1990). A recent outbreak of dengue fever in north Queensland. Commun. Dis. Intell..

[19] McBride W.J.H., Mullner H., LaBrooy J.T., Wronski I. (1998). The 1993 dengue 2 epidemic in Charters Towers, north Queensland: Clinical features and public health impact. Epidemiol. Infect..

[20] Row D., Pearce M., Hapgood G., Sheridan J. (1993). Dengue and dengue haemorrhagic fever in Charters Towers, Queensland. Commun. Dis. Intell..

[21] Streatfield R., Sinclair D.P., Bielby G., Sheridan J., Pearce M., Phillips D. (1993). Dengue serotye 2 epidemic Townsville, 1992–93. Commun. Dis. Intell..

[22] Ritchie S., Hanna J., van den Hurk A., Harley D., Lawrence R., Phillips D. (1995). Importation and subsequent local transmission of dengue 2 in Cairns. Commun. Dis. Intell..

[23] Hanna J.N., Ritchie S.A. (2009). Outbreaks of dengue in north Queensland, 1990–2008. Commun. Dis. Intell..

[24] Hanna J.N., Ritchie S.A., Merritt A.D., van den Hurk A.F., Phillips D.A., Serafin I.L., Norton R.E., McBride W.J.H., Gleeson F.V., Podinger M. (1998). Two contiguous outbreaks of dengue type 2 in north Queensland. Med. J. Aust..

[25] Cleland J.B., Bradley B. (1918). Dengue fever in Australia: Its history and clinical course, its experimental transmission by *Stegomyia fasciata*, and the results of inoculation and other experiments. J. Hyg..

[26] Cleland J.B., Bradley B., Macdonald W. (1919). Further experiments in the etiology of dengue fever. J. Hyg..

[27] Cleland J.B., Bradley B., McDonald W. (1916). On the transmission of Australian dengue by the mosquito *Stegomyia fasciata*. Med. J. Aust..

[28] Pyke A.T. (2018). The origins of dengue outbreaks in northern Queensland, Australia, 1990–2017. Microbiol. Aust..

[29] Ritchie S.A., Hanna J.N., Hills S.L., Piispanen J.P., McBride W.J.H., Pyke A., Spark R.L. (2002). Dengue control in north Queensland, Australia: Case recognition and selective indoor residual spraying. Dengue Bull..

[30] Malcolm R.L., Hanna J.N., Phillips D.A. (1999). The timeliness of notification of clinically suspected cases of dengue fever imported into north Queensland. Aust. N.Z. J. Public Health.

[31] Graham H. (1903). The dengue: A study of its pathology and mode of propagation. J. Trop. Med..

[32] Bancroft T.L. (1906). On the etiology of dengue fever. Aust. Med. Gaz..

[33] Theobald F.V. (1901). A Monograph of the Culicidae, or Mosquitoes. Vol. 1.

[34] Siler J.F., Hall M.W., Hitchens A.P. (1925). Results obtained in the transmission of dengue fever. J. Am. Med. Assoc..

[35] Achee N.L., Gould F., Perkins T.A., Reiner R.C., Morrison A.C., Ritchie S.A., Gubler D.J., Teyssou R., Scott T.W. (2015). A critical assessment of vector control for dengue prevention. PLoS Negl. Trop. Dis..

[36] Bowman L.R., Runge-Ranzinger S., McCall P.J. (2014). Assessing the relationship between vector indices and dengue transmission: A systematic review of the evidence. PLoS Negl. Trop. Dis..

[37] Tun-Lin W., Kay B.H., Barnes A. (1995). Understanding productivity, a key to *Aedes aegypti* surveillance. Am. J. Trop. Med. Hyg..

[38] Kay B.H., Ryan P.A., Russell B.M., Holt J.S., Lyons S.A., Foley P.N. (2000). The importance of subterranean mosquito habitat to arbovirus vector control strategies in north Queensland, Australia. J. Med. Entomol..

[39] Russell B.M., McBride W.J., Mullner H., Kay B.H. (2002). Epidemiological significance of subterranean *Aedes aegypti* (Diptera: Culicidae) breeding sites to dengue virus infection in Charters Towers, 1993. J. Med. Entomol..

[40] Montgomery B.L., Ritchie S.A. (2002). Roof gutters: A key container for *Aedes aegypti* and *Ochlerotatus notoscriptus* (Diptera: Culicidae) in Australia. Am. J. Trop. Med. Hyg..

[41] Ritchie S.A., Long S., Hart A., Webb C.E., Russell R.C. (2003). An adulticidal sticky ovitrap for sampling container-breeding mosquitoes. J. Am. Mosq. Control. Assoc..

[42] Ritchie S.A., Long S., Smith G., Pyke A., Knox T.B. (2004). Entomological investigations in a focus of dengue transmission in Cairns, Queensland, Australia, by using the sticky ovitraps. J. Med. Entomol..

[43] Williams C.R., Long S.A., Russell R.C., Ritchie S.A. (2006). Field efficacy of the BG-sentinel compared with CDC backpack aspirators and CO_2_-baited EVS traps for collection of adult *Aedes aegypti* in Cairns, Queensland, Australia. J. Am. Mosq. Control. Assoc..

[44] Williams C.R., Long S.A., Webb C.E., Bitzhenner M., Geier M., Russell R.C., Ritchie S.A. (2007). *Aedes aegypti* population sampling using BG-Sentinel traps in north Queensland Australia: Statistical considerations for trap deployment and sampling strategy. J. Med. Entomol..

[45] Metzger M.E., Hardstone Yoshimizu M., Padgett K.A., Hu R., Kramer V.L. (2017). Detection and establishment of *Aedes aegypti* and *Aedes albopictus* (Diptera: Culicidae) mosquitoes in California, 2011–2015. J. Med. Entomol..

[46] Scholte E., Den Hartog W., Dik M., Schoelitsz B., Brooks M., Schaffner F., Foussadier R., Braks M., Beeuwkes J. (2010). Introduction and control of three invasive mosquito species in The Netherlands, July–October 2010. Euro Surveill..

[47] Eiras A.E., Buhagiar T.S., Ritchie S.A. (2014). Development of the Gravid *Aedes* Trap for the capture of adult female container-exploiting mosquitoes (Diptera: Culicidae). J. Med. Entomol..

[48] Ritchie S.A., Buhagiar T.S., Townsend M., Hoffmann A., van den Hurk A.F., McMahon J.L., Eiras A.E. (2014). Field validation of the Gravid *Aedes* Trap (GAT) for collection of *Aedes aegypti* (Diptera: Culicidae). J. Med. Entomol..

[49] Johnson B.J., Ritchie S.A. (2016). The siren’s song: Exploitation of female flight tones to passively capture male *Aedes aegypti* mosquitoes. J. Med. Entomol..

[50] Queensland Health (1994). A Dengue Fever Management Plan for North Queensland.

[51] Reiter P., Gubler D.J., Gubler D.J., Kuno G. (1997). Surveillance and control of urban dengue vectors. Dengue and Dengue Hemorrhagic Fever.

[52] Henrick C.A. (2007). Methoprene. J. Am. Mosq. Control. Assoc..

[53] Hanna J.N., Ritchie S.A., Phillips D.A., Serafin I.L., Hills S.L., van den Hurk A.F., Pyke A.T., McBride W.J.H., Amadio M.G. (2001). An epidemic of dengue 3 in far north Queensland, 1997–1999. Med. J. Aust..

[54] Vazquez-Prokopec G.M., Montgomery B.L., Horne P., Clennon J.A., Ritchie S.A. (2017). Combining contact tracing with targeted indoor residual spraying significantly reduces dengue transmission. Sci. Adv..

[55] Perich M.J., Kardec A., Braga I.A., Portal I.F., Burge R., Zeichner B.C., Brogdon W.A., Wirtz R.A. (2003). Field evaluation of a lethal ovitrap against dengue vectors in Brazil. Med. Vet. Entomol..

[56] Sithiprasasna R., Mahapibul P., Noigamol C., Perich M.J., Zeichner B.C., Burge B., Norris S.L., Jones J.W., Schleich S.S., Coleman R.E. (2003). Field evaluation of a lethal ovitrap for the control of *Aedes aegypti* (Diptera: Culicidae) in Thailand. J. Med. Entomol..

[57] Ritchie S.A. (2005). Evolution of dengue control strategies in north Queensland, Australia. Arbovirus Res. Aust..

[58] Vazquez-Prokopec G.M., Kitron U., Montgomery B., Horne P., Ritchie S.A. (2010). Quantifying the spatial dimension of dengue virus epidemic spread within a tropical urban environment. PLoS Negl. Trop. Dis..

[59] Endersby-Harshman N.M., Wuliandari J.R., Harshman L.G., Frohn V., Johnson B.J., Ritchie S.A., Hoffmann A.A. (2017). Pyrethroid susceptibility has been maintained in the dengue vector, *Aedes aegypti* (Diptera: Culicidae), in Queensland, Australia. J. Med. Entomol..

[60] Kay B., Nam V.S. (2005). New strategy against *Aedes aegypti* in Vietnam. Lancet.

[61] Jennings C.D., Phommasack B., Sourignadeth B., Kay B.H. (1995). *Aedes aegypti* control in the Lao People’s Democratic Republic, with reference to copepods. Am. J. Trop. Med. Hyg..

[62] Riviere F., Kay B.H., Klein J.M., Sechan Y. (1987). *Mesocyclops aspericornis* (Copepoda) and *Bacillus thuringiensis* var. israelensis for the biological control of Aedes and Culex vectors (Diptera: Culicidae) breeding in crab holes, tree holes, and artificial containers. J. Med. Entomol..

[63] Russell B.M., Muir L.E., Weinstein P., Kay B.H. (1996). Surveillance of the mosquito *Aedes aegypti* and its biocontrol with the copepod *Mesocyclops aspericornis* in Australian wells and gold mines. Med. Vet. Entomol.

[64] Kay B.H., Cabral C.P., Araujo D.B., Ribeiro Z.M., Braga P.H., Sleigh A.C. (1992). Evaluation of a funnel trap for collecting copepods and immature mosquitoes from wells. J. Am. Mosq. Control. Assoc..

[65] Knox T.B., Yen N.T., Vu S.N., Gatton M.L., Kay B.H., Ryan P.A. (2007). Critical evaluation of quantitative sampling methods for *Aedes aegypti* (Diptera: Culicidae) immatures in water storage containers in Vietnam. J. Med. Entomol..

[66] Vu S.N., Nguyen T.Y., Kay B.H., Marten G.G., Reid J.W. (1998). Eradication of *Aedes aegypti* from a village in Vietnam, using copepods and community participation. Am. J. Trop. Med. Hyg..

[67] Kay B.H., Vu S.N., Tien T.V., Yen N.T., Phong T.V., Diep V.T., Ninh T.U., Bektas A., Aaskov J.G. (2002). Control of *Aedes* vectors of dengue in three provinces of Vietnam by use of *Mesocyclops* (Copepoda) and community-based methods validated by entomologic, clinical, and serological surveillance. Am. J. Trop. Med. Hyg..

[68] Vu S.N., Thi Yen N., Minh Duc H., Cong Tu T., TrongThang V., Hoang Le N., Hoang San L., Le Loan L., QueHuong V.T., Kim Khanh L.H. (2012). Community-based control of *Aedes aegypti* by using *Mesocyclops* in southern Vietnam. Am. J. Trop. Med. Hyg..

[69] Kay B.H., TuyetHanh T.T., Le N.H., Quy T.M., Vu S.N., Hang P.V., Yen N.T., Hill P.S., Vos T., Ryan P.A. (2010). Sustainability and cost of a community-based strategy against *Aedes aegypti* in northern and central Vietnam. Am. J. Trop. Med. Hyg..

[70] McGraw E.A., O’Neill S.L. (2013). Beyond insecticides: New thinking on an ancient problem. Nat. Rev. Microbiol..

[71] Ritchie S.A., van den Hurk A.F., Smout M.J., Staunton K.M., Hoffmann A.A. (2018). Mission accomplished? We need a guide to the ‘post-release’ world of wolbachia for *Aedes*-borne disease control. Trends Parasitol..

[72] Werren J.H., Baldo L., Clark M.E. (2008). *Wolbachia*: Master manipulators of invertebrate biology. Nat. Rev. Microbiol..

[73] McMeniman C.J., Lane A.M., Fong A.W., Voronin D.A., Iturbe-Ormaetxe I., Yamada R., McGraw E.A., O’Neill S.L. (2008). Host adaptation of a *Wolbachia* strain after long-term serial passage in mosquito cell lines. Appl. Environ. Microbiol..

[74] Xi Z., Khoo C.C., Dobson S.L. (2005). *Wolbachia* establishment and invasion in an *Aedes aegypti* laboratory population. Science.

[75] McMeniman C.J., Lane R.V., Cass B.N., Fong A.W., Sidhu M., Wang Y.F., O’Neill S.L. (2009). Stable introduction of a life-shortening *Wolbachia* infection into the mosquito *Aedes aegypti*. Science.

[76] McMeniman C.J., O’Neill S.L. (2010). A virulent *Wolbachia* infection decreases the viability of the dengue vector *Aedes aegypti* during periods of embryonic quiescence. PLoS Negl. Trop. Dis..

[77] Turley A.P., Moreira L.A., O’Neill S.L., McGraw E.A. (2009). *Wolbachia* infection reduces blood-feeding success in the dengue fever mosquito, *Aedes aegypti*. PLoS Negl. Trop. Dis..

[78] Hedges L.M., Brownlie J.C., O’Neill S.L., Johnson K.N. (2008). *Wolbachia* and virus protection in insects. Science.

[79] Moreira L.A., Iturbe-Ormaetxe I., Jeffery J.A., Lu G., Pyke A.T., Hedges L.M., Rocha B.C., Hall-Mendelin S., Day A., Riegler M. (2009). A *Wolbachia* symbiont in *Aedes aegypti* limits infection with dengue, chikungunya, and *Plasmodium*. Cell.

[80] Hoffmann A.A., Montgomery B.L., Popovici J., Iturbe-Ormaetxe I., Johnson P.H., Muzzi F., Greenfield M., Durkan M., Leong Y.S., Dong Y. (2011). Successful establishment of *Wolbachia* in *Aedes* populations to suppress dengue transmission. Nature.

[81] Yeap H.L., Mee P., Walker T., Weeks A.R., O’Neill S.L., Johnson P., Ritchie S.A., Richardson K.M., Doig C., Endersby N.M. (2011). Dynamics of the ‘popcorn’ *Wolbachia* infection in outbred *Aedes aegypti* informs prospects for mosquito vector control. Genetics.

[82] Walker T., Johnson P.H., Moreira L.A., Iturbe-Ormaetxe I., Frentiu F.D., McMeniman C.J., Leong Y.S., Dong Y., Axford J., Kriesner P. (2011). The *w*Mel *Wolbachia* strain blocks dengue and invades caged *Aedes aegypti* populations. Nature.

[83] Ritchie S.A., Johnson P.H., Freeman A.J., Odell R.G., Graham N., Dejong P.A., Standfield G.W., Sale R.W., O’Neill S.L. (2011). A secure semi-field system for the study of *Aedes aegypti*. PLoS Negl. Trop. Dis..

[84] Schmidt T.L., Barton N.H., Rasic G., Turley A.P., Montgomery B.L., Iturbe-Ormaetxe I., Cook P.E., Ryan P.A., Ritchie S.A., Hoffmann A.A. (2017). Local introduction and heterogeneous spatial spread of dengue-suppressing *Wolbachia* through an urban population of *Aedes aegypti*. PLoS Biol..

[85] Frentiu F.D., Zakir T., Walker T., Popovici J., Pyke A.T., van den Hurk A., McGraw E.A., O’Neill S.L. (2014). Limited dengue virus replication in field-collected *Aedes aegypti* mosquitoes infected with *Wolbachia*. PLoS Negl. Trop. Dis..

[86] Carrington L.B., Tran B.C.N., Le N.T.H., Luong T.T.H., Nguyen T.T., Nguyen P.T., Nguyen C.V.V., Nguyen H.T.C., Vu T.T., Vo L.T. (2018). Field- and clinically derived estimates of *Wolbachia*-mediated blocking of dengue virus transmission potential in *Aedes aegypti* mosquitoes. Proc. Natl. Acad. Sci. USA.

[87] Nguyen T.H., Nguyen H.L., Nguyen T.Y., Vu S.N., Tran N.D., Le T.N., Vien Q.M., Bui T.C., Le H.T., Kutcher S. (2015). Field evaluation of the establishment potential of *w*MelPop *Wolbachia* in Australia and Vietnam for dengue control. Parasites Vectors.

[88] Ritchie S.A., Townsend M., Paton C.J., Callahan A.G., Hoffmann A.A. (2015). Application of *w*MelPop *Wolbachia* strain to crash local populations of *Aedes aegypti*. PLoS Negl. Trop. Dis..

[89] Dorigatti I., McCormack C., Nedjati-Gilani G., Ferguson N.M. (2018). Using *Wolbachia* for dengue control: Insights from modelling. Trends Parasitol..

[90] Lambrechts L., Ferguson N.M., Harris E., Holmes E.C., McGraw E.A., O’Neill S.L., Ooi E.E., Ritchie S.A., Ryan P.A., Scott T.W. (2015). Assessing the epidemiological effect of *Wolbachia* for dengue control. Lancet Infect. Dis..

[91] Anders K.L., Indriani C., Ahmad R.A., Tantowijoyo W., Arguni E., Andari B., Jewell N.P., Rances E., O’Neill S.L., Simmons C.P. (2018). The AWED trial (Applying *Wolbachia* to Eliminate Dengue) to assess the efficacy of *Wolbachia*-infected mosquito deployments to reduce dengue incidence in Yogyakarta, Indonesia: Study protocol for a cluster randomised controlled trial. Trials.

[92] Dodson B.L., Hughes G.L., Paul O., Matacchiero A.C., Kramer L.D., Rasgon J.L. (2014). *Wolbachia* enhances West Nile virus (WNV) infection in the mosquito *Culex tarsalis*. PLoS Negl. Trop. Dis..

[93] Ant T.H., Herd C.S., Geoghegan V., Hoffmann A.A., Sinkins S.P. (2018). The *Wolbachia* strain *w*Au provides highly efficient virus transmission blocking in *Aedes aegypti*. PLoS Pathog..

[94] Joubert D.A., Walker T., Carrington L.B., De Bruyne J.T., Kien D.H., Hoang Nle T., Chau N.V., Iturbe-Ormaetxe I., Simmons C.P., O’Neill S.L. (2016). Establishment of a *Wolbachia* superinfection in *Aedes aegypti* mosquitoes as a potential approach for future resistance management. PLoS Pathog..

[95] Soper F.L. (1965). The 1964 status of *Aedes aegypti* eradication and yellow fever in the Americas. Am. J. Trop. Med. Hyg..

[96] Communicable Diseases Branch, Queensland Health (2018). Mosquito-Borne Diseases in Queensland, 1 July 2012–30 June 2017.

